# A Study to Analyze Refractive Errors in Relation to Age and Sex

**DOI:** 10.7759/cureus.37834

**Published:** 2023-04-19

**Authors:** Hassan Mohammad, Wajid A Chatha, Maha M Ahmed Abdul-Latif, Nada M Hakem Al-Mijlad

**Affiliations:** 1 Anatomy, College of Medicine/Northern Border University, Arar, SAU; 2 Ophthalmology, College of Medicine/Northern Border University, Arar, SAU; 3 Dermatology, University Health Center/Northern Border University, Arar, SAU

**Keywords:** spherical equivalent, hyperopia, high myopia, refractive error, age and sex

## Abstract

Background and objective

Refractive defects should be detected and treated early to avoid irreversible vision loss and other potential problems in the future. In this study, we aimed to analyze the refractive errors (REs) and their relationship with gender and age.

Methods

This study was conducted at the Northern Border University Health Center, Arar, Saudi Arabia. REs were analyzed using spherical equivalents (SEs), cylinders, and their orientations. SEs of REs were taken as half the cylinder plus the spherical component. Emmetropia was defined as SE between -0.50 and +0.50 diopter sphere (DS), myopia as SE ≤0.50 DS, and hyperopia as SE ≥0.50 DS for adults and SE ≥1.0 for children (up to 10 years). Statistical analysis was performed using the IBM SPSS Statistics software package (IBM, Armonk, NY). Qualitative data were presented as frequency and percentage while quantitative data were presented as mean and standard deviation (SD). Chi-square was used as a significant test and a p-value <0.05 was considered statistically significant.

Results

A total of 240 patients were included in the study. There were 138 men and 102 females aged 3-60 years (57.5 and 42.5%, respectively). The mean age of males was 24.4 years and that of females was 25.5 years. The p-value was statistically significant in terms of analysis with age. The study found an association between age and RE magnitude and variability.

Conclusion

Based on our findings, RE is a common problem that affects individuals of all ages. Regular screenings are advised for individuals in order to detect REs early.

## Introduction

Refractive error (RE) is one of the most common eye diseases in children and adolescents and one of the biggest public health problems globally. It has been reported that 42% of visual impairment worldwide is due to RE [1]. RE has a profound effect on children, as it not only increases the possibility of pathological eye changes such as myopic macular degeneration and retinal degeneration, which can lead to irreversible blindness, but also seriously affects the psychosocial well-being of children, which may limit their educational opportunities and outcomes [2-4]. REs occur when the eye does not focus light correctly on the retina, resulting in blurred vision. This is caused by the eye growing too long (myopia), the eye not growing enough (hyperopia), the shape of the cornea not focusing evenly (astigmatism), or an age-related lack of near vision (presbyopia). Light rays usually travel in parallel lines from distant objects (6 meters and beyond) and upon contact with an emmetropic (normal) eye, they are sharply focused on the retina (film of the eye), forming a sharp film image of a distant object without the eye having to activate its adaptive (focusing) system to fine-tune the image. However, in most eyes, the image does not focus accurately on the retina when the adaptive system is relaxed. This eye disease is called RE. It is further classified according to where the image is focused. A review of the literature and medical databases reveals that since 1990, many studies on the epidemiology of REs have been conducted worldwide [5,6]. The number of cases of visual impairment and blindness is expected to increase significantly as the general burden of disease shifts to non-communicable diseases and disabilities as a result of demographic and developmental changes in the population [7].

RE is an established and serious public health concern. RE, especially myopia, is a common problem among young people, especially students. Also, presbyopia is very common in people aged 40 years and older. A student may lose interest in learning if spelling mistakes are not corrected. Likewise, working people and the elderly can suffer from a lack of RE correction. This, in turn, can cause educational loss, economic losses, and reduced quality of life [8]. Several studies have demonstrated that uncorrected REs account for a sizable portion of vision impairment [9,10].

There is evidence to suggest that myopia is reaching epidemic proportions at a rate that indicates a strong environmental influence [11]. Myopia is not only an optical disability, but its detrimental effect on the eyeball itself includes multiple risks for many sight-threatening eye diseases, including cataracts, glaucoma, retinal detachment, and myopic retinopathy [12]. Eye diseases usually appear in childhood or older adulthood; consequently, most studies on eye diseases and RE have involved children or older adults. On the other hand, scarce attention has been paid to the eye health of young adults in the literature [13].

Considering the general assessment of the magnitude of RE and its trends over time, we perform this analysis to analyze REs, which can provide useful information. The main objective of this study was to find out if there is any significant association of RE with age and sex among patients at the Northern Border University Health Center.

## Materials and methods

The patients’ data were collected from the ophthalmology department, University Health Centre, Northern Border University, Arar, Saudi Arabia for this retrospective study. The data included patients' age, sex, and digital auto-refractometer report. The data of patients who visited the hospital from January 2021 to November 2022 were collected. Descriptive statistics [mean, standard deviation (SD)] were used to characterize the data. A total of 240 patients belonging to different age groups were included in the study. According to age, they were classified into four groups: Group A: patients aged 3-10 years; Group B: patients aged 11-20 years; Group C: patients aged 21-40 years; Group D: patients aged 41-60 years.

REs were analyzed using spherical equivalents (SEs), cylinders, and their orientations. The patients visited the health center with symptoms such as double vision, hazy vision, seeing glare or halo around bright light, squinting, headache, eye strain, and trouble focusing while reading or looking at the computer. Then the ophthalmologists advised them to test their eyes with a digital auto-refractometer. The report of the digital auto-refractometer tests shows the measurements of the sphere, cylinder, axial, SEs, and pupillary distance of both eyes. The pupillary distance was excluded from the study. SEs of REs were taken as half the cylinder plus the spherical component. Emmetropia was defined as SE between -0.50 and +0.50 diopter sphere (DS), myopia as SE ≤0.50 DS, and hyperopia as SE ≥0.50 DS for adults and SE ≥1.0 for children (up to 10 years). Individuals were considered myopic if one or both eyes were myopic, and hyperopic if one or both eyes were hyperopic. Statistical analysis was performed using the IBM SPSS Statistics software package (IBM, Armonk, NY). Qualitative data were presented as frequency and percentage while quantitative data were presented as mean and standard deviation (SD). Chi-square was used as a significant test and a p-value <0.05 was considered statistically significant. Ethical approval was granted by the Local Committee of Bioethics (HAP-09-A-043) at the Northern Border University (decision no: 23/44/H dated 02/02/2023).

## Results

The patient data included age, sex, and digital auto-refractometer reports of those who visited the center from January 2021 to November 2022; 240 patients were included in the study. There were 138 men and 102 females aged 3-60 years (57.5 and 42.5% respectively) (Table 1). The mean age of males was 24.4 (SD: 17.38) years and that of females was 25.5 (SD: 17.70) years. Group A had the highest proportion of male patients (36%) while Group B (16.7%) had the lowest. Group B had the highest proportion of female patients (28.4%) while Group C (22.5%) had the lowest. Group A had 76 (31.7%) patients [50 (36.2%) male patients and 26 (25.5%) female patients]. Group B had 52 (21.7%) patients [23 (16.7%) male patients and 29 (28.4%) female patients]. Group C had 48 (20%) patients [25 (18.1%) male patients and 23 (22.5%) female patients]. Group D had 64 (26.7%) patients [40 (29%) male patients and 24 (23.5%) female patients].

**Table 1:** Age and sex distribution of the study cohort

Age group, years	Male, n (%)	Female, n (%)	P-value
3-10 (Group A)	50 (36.2%)	26 (25.5%)	0.068
11-20 (Group B)	23 (16.7%)	29 (28.4%)	
21-40 (Group C)	25 (18.1%)	23 (22.5%)	
41-60 (Group D)	40 (29%)	24 (23.5%)	
Total	138 (57.5%)	102 (42.5%)	

The right eye measurements in males were as follows: 51 (37%) patients were normal, 65 (47%) patients were myopic, and 22 (16%) patients were hyperopic. Regarding the right eye analysis in females, 34 (33.3%) patients were normal, 51 (50%) patients were myopic, and 17 (16.6%) patients were hyperopic. The right eye measurements for both males and females were as follows: 85 (35.4%) normal patients, 116 (48.3%) myopic patients, and 39 (16.3%) hyperopic patients (Table 2). The right eye analysis was statistically significant in terms of age (Table 3). Group B had the most number of myopic patients while Group D had the least. Group A had the most number of hyperopic patients while Group C had the least. Group A had the most number of normal patients while Group B had the least. Hyperopia was statistically significant in group A (p=0.00).

**Table 2:** Right eye analysis with respect to sex

Sex		Normal, n (%)	Myopia, n (%)	Hyperopia, n (%)	Total, n (%)
	Male	51 (37%)	65 (47%)	22 (16%)	138 (57.5%)
	Female	34 (33.3%)	51 (50%)	17 (16.6%)	102 (42.5%)
Total		85 (35.4%)	116 (48.3%)	39 (16.3%)	240 (100%)

**Table 3:** Right eye analysis with respect to age

	Group A, n (%)	Group B, n (%)	Group C, n (%)	Group D, n (%)	P-value
Emmetropia	32 (37.8%)	10 (11.8%)	12 (14.1%)	31 (36.5%)	0.00
Myopia	27 (23.3%)	36 (31%)	31 (26.7%)	22 (19%)	
Hyperopia	17 (43.6%)	6 (15.4%)	5 (12.8%)	11 (28.2%)	
Total	76 (31.7%)	52 (21.7%)	48 (20%)	64 (26.7%)	

The left eye measurements in males were as follows: 54 (69.2%) patients were normal, 57 (50%) patients were myopic, and 27 (56.3%) patients were hyperopic. The left eye analyses in females were as follows: 24 (30.8%) patients were normal, 57 (50%) patients were myopic, and 21 (43.8%) patients were hyperopic. The left eye measurements for both males and females were as follows: 78 (32.5%) normal patients, 114 (47.5%) myopic patients, and 48 (20%) hyperopic patients. The left eye analysis was statistically significant in terms of both age and sex (Tables 4-5). Group A had the most number of hyperopic patients, Group B had the most number of myopic patients, and Group D had the most number of normal patients. Of note, 50% of males and 50% of females were myopic; 56.3% of males were hyperopic and the difference was statistically significant (p=0.29).

**Table 4:** Left eye analysis with respect to sex

Sex		Normal, n (%)	Myopia, n (%)	Hyperopia, n (%)	Total, n (%)	P-value
	Male	54 (69.2%)	57 (50%)	27 (56.3%)	138 (57.5%)	0.029
	Female	24 (30.8%)	57 (50%)	21 (43.8%)	102 (42.5%)	
Total		78 (32.5%)	114 (47.5%)	48 (20%)	240 (100%)	

**Table 5:** Left eye analysis with respect to age

	Group A, n (%)	Group B, n (%)	Group C, n (%)	Group D, n (%)	P-value
Emmetropia	25 (32.1%)	12 (15.4%)	14 (17.9%)	27 (34.6%)	0.022
Myopia	31 (27.2%)	33 (28.9%)	28 (24.6%)	22 (19.3%)	
Hyperopia	20 (41.7%)	7 (14.6%)	6 (12.5%)	15 (31.3%)	
Total	76 (31.7%)	52 (21.7%)	48 (20%)	64 (26.7%)	

## Discussion

The distribution of RE in different age groups showed age-related changes, an important aspect in terms of determining the public health impact of RE severity and understanding the mechanisms governing the development of refractive ability. A study conducted in Saudi Arabia showed that the majority of 12- to 13-year-old children had an RE between -0.50 and -3.00 DS [14]. Refraction is an important part of the vision development process and the condition of the refractive image changes with age. If the eye has an RE, light from a distant object is focused either in front of or behind the retina. Feedback related to visual input is generally considered to be a central mechanism in the development of REs. Refractive development usually begins in both eyes in a hyperopic state, and the growth of the eyeball is controlled by feedback from both the retina and the visual brain, which processes binocular information [15-17].

We found that males had higher myopia and hyperopia. Compared to men, women [18] had different degrees of myopia [19]. An epidemiological review of myopia found that women had a higher prevalence than men. The severity of myopia varies with age, with most cases occurring between the ages of one and 10 years and remaining relatively stable between the ages of 12 and 50 years [20]. In developed nations, myopia is the most frequent cause of treatable visual impairment in both adults and children [21-25], and it is a major factor in avoidable blindness in developing nations [26]. Myopia affects roughly one in six people worldwide [27]. This represents a significant global burden, particularly in poorer nations where there is a significant unmet need for visual correction [28]. The radiating fibers of the suspensory ligament relax when the ciliary muscle contracts and moves the ciliary body forward and inward to accommodate the eye for near objects. As a result, the elastic lens might take on a globular form. Getting older causes the lens to become thicker and less elastic, which reduces its capacity to accommodate (presbyopia). The synchronized contraction of the medial rectus muscles causes the convergence of the eyes during lens accommodation. A precise knowledge of the RE and its relationship to age and sex is necessary to prevent vision loss.

In our study, in all groups (A-D), myopia was found to be more prevalent than hyperopia. The high number of REs in this study may be attributed to particular lifestyles or living conditions. Visual efficiency, which describes the quality of efficiency in the data acquisition process, depends on an individual's ability to focus and approach, as well as eye movements. Poor concentration significantly affects learning and reading. Thus, an eye examination among individuals is necessary to rule out the presence of REs, many of which require therapy, while others require ongoing monitoring to manage the individual's age-related deterioration. In addition, REs should be treated as early as possible to reduce possible adverse effects. The results of this study are relevant to the teaching and research in the field of epidemiology. The results can guide clinical optometrists on the types of REs, expected frequencies, and prescription intervals for different age groups and sexes. Overall, the results of this study show an association of age with the magnitude and variability of RE.

## Conclusions

RE is a common and significant cause of visual impairment. The results of this study showed the extent and pattern of RE among patients attending the Department of Ophthalmology at the Northern Border University Health Center. In our study, myopia and hyperopia were more common among men. Myopia was found to be more prevalent than hyperopia in all groups (A-D). Regular collection of statistics on the extent and pattern of REs will help inform plans to combat this common disorder and its complications in the community and country.
